# Investigation of Responsiveness to Thyrotropin-Releasing Hormone in Growth Hormone-Producing Pituitary Adenomas

**DOI:** 10.1155/2013/159858

**Published:** 2013-11-21

**Authors:** Sang Ouk Chin, Sang Youl Rhee, Suk Chon, You-Cheol Hwang, In-Kyung Jeong, Seungjoon Oh, Sung-Woon Kim

**Affiliations:** Department of Endocrinology and Metabolism, Kyung Hee University School of Medicine, 23 Kyungheedae-ro, Dongdaemoon-gu, Seoul 130-702, Republic of Korea

## Abstract

*Objective*. The aim of this study was to investigate how the paradoxical response of GH secretion to TRH changes according to tumor volumes. *Methods*. Patients with newly diagnosed acromegaly were classified as either TRH responders or nonresponders according to the results of a TRH stimulation test (TST), and their clinical characteristics were compared according to responsiveness to TRH and tumor volumes. *Results*. A total of 41 acromegalic patients who underwent the TST were included in this study. Between TRH responders and nonresponders, basal GH, IGF-I levels, peak GH levels, and tumor volume were not significantly different, but the between-group difference of GH levels remained near significant over the entire TST time. ΔGH_max-min_ during the TST were significantly different according to the responsiveness to TRH. Peak GH levels and ΔGH_max-min_ during the TST showed significantly positive correlations with tumor volume with higher levels in macroadenomas than in microadenomas. GH levels over the entire TST time also remained significantly higher in macroadenomas than in microadenomas. *Conclusion*. Our data demonstrated that the paradoxical response of GH secretion to TRH in GH-producing pituitary adenomas was not inversely correlated with tumor volumes.

## 1. Introduction

Abnormal responsiveness of growth hormone- (GH-) producing pituitary adenomas to hypothalamic hormones has been previously described [1]. This paradoxical response of GH to thyrotropin-releasing hormone (TRH) in GH-producing pituitary adenomas was first reported in 1972 [2, 3] and observed in 50~75% of untreated acromegalic patients [4]. However, this response is not specific to acromegaly and was also found in various pathologic conditions such as severe hepatic failure [2], chronic renal failure [5], diabetes mellitus [6], and anorexia nervosa [7]. 

There have been many studies of the predictive value of the paradoxical response for treatment outcome and prognosis in acromegaly [4, 8–10]. However, the detailed mechanism of the paradoxical response of GH to TRH in acromegaly remains unknown despite a number of possible hypotheses: local production of TRH by adenoma cells [11, 12], TRH-induced release of GH [13], TRH production by anterior pituitary gland [14], and inappropriate expression of TRH receptors at tumor cells [15]. Moreover, only a few studies have attempted to observe how the pattern of paradoxical response changes according to tumor volume [10, 16]. The aim of this study was to investigate how the paradoxical response of GH secretion to TRH changes according to tumor volume in acromegalic patients. 

## 2. Methods

### 2.1. Patients

A total of 65 patients newly diagnosed with acromegaly at Kyung Hee University Hospital between 2005 and 2012 were initially screened. All of them were diagnosed with acromegaly when an oral glucose tolerance test (OGTT) failed to suppress GH levels below 1.0 *μ*g/L, and their insulin-like growth factor I (IGF-I) level was above the upper normal range for age. Among these patients, 41 patients (25 men and 16 women) who underwent the TRH stimulation test (TST) during the diagnostic work-up procedure for acromegaly were enrolled. Their mean age was 41 ± 11 yr. None of these patients received any medications for acromegaly such as somatostatin analogues before the surgical treatment. 

### 2.2. Oral Glucose Tolerance Test

The OGTT was performed after an overnight fast. Patients had blood samples taken at baseline (0 minutes) and then at 30, 60, 90, and 120 minutes after drinking 75 g of a glucose solution. Blood was allowed to clot at room temperature for 15 minutes and then was centrifuged; the serum was frozen at −80°C in multiple aliquots. Blood samples from all time points were assayed for GH levels, and baseline samples were also assayed for IGF-I.

### 2.3. TRH Stimulation Test

Patients had blood samples taken at baseline (0 minute) and then at 30, 60, 90, and 120 minutes after intravenous administration of 200 *μ*g TRH [10]. TRH-induced GH responsiveness was evaluated by calculating the TRH ratio (the peak/basal ratio of GH during the TST): those with a TRH ratio higher than 2 was defined as TRH responders. This definition had been used in previous reports [16–19]. The assays for GH during the TST were performed in the same way as during the OGTT.

### 2.4. Octreotide Suppression Test

After an overnight fast, all patients had baseline blood samples taken for GH, and 100 *μ*g of octreotide (Sandostatin, Novartis) was then administered intravenously. Blood sampling for GH testing was continued every hour for four hours. Responders to the OST were defined as those whose nadir GH level was less than 2.5 *μ*g/L during the test [20]. The assays for GH during the TST were performed in the same way as during the OGTT.

### 2.5. Image Work-Up

Pituitary adenomas were identified in all patients by sellar MRI. Microadenoma was defined as an intrasellar tumor with a diameter less than 10 mm and macroadenoma as a tumor having a diameter greater than 10 mm and impinging upon adjacent sellar structures. The tumor volume was estimated by the equation which had been developed by Di Chiro and Nelson (volume = 0.5 × length × height × width) [21]. 

### 2.6. Measurement of GH and IGF-I

Serum GH concentration was measured using commercial radioimmunoassay (RIA) kits (hGH-RIACT, Cisbio Bioassays, Bedford, MA, USA). The sensitivity of this kit was 0.01 *μ*g/L with an intra-assay CV of 3.8–5.0% and an interassay CV of 1.3–2.1%. Serum IGF-I concentrations were determined by commercial immunoradiometric assay kits (IGF-I NEXT IRMA CT, IDA S.A., Liège, Belgium). The minimum detectable concentration of IGF-I was 1.25 *μ*g/L. The intra- and interassay CVs were 2.6–4.4% and 7.4–9.1%, respectively. 

### 2.7. Data Analysis

Clinical characteristics of patients were compared according to their responsiveness to TRH, which included age, body mass index (BMI), basal GH and IGF-I, peak GH levels and ΔGH_max-min_ (defined as the difference between the peak and basal GH levels) during the TST, and tumor volume. GH levels during 120 minutes of the entire TST time were also compared between TRH responders and nonresponders. Then, the same analyses were conducted between macroadenomas and microadenomas. Lastly, the correlation coefficients to explore the relationship between responsiveness to TRH and tumor volumes were calculated. In addition, the ratios between the basal and peak GH levels during the OST were calculated and compared according to the responsiveness to TRH in order to investigate how GH suppression rates during the OST would be different between TRH responders and nonresponders.

### 2.8. Statistical Analysis

All statistical analyses were performed with PASW (version 18.0; SPSS Inc., Chicago, IL, USA), and a *P* value < 0.05 was considered statistically significant. Baseline characteristics were described as means and standard deviations. The Mann-Whitney test and repeated measure one-way analysis of variance (RMANOVA) were used to compare the data according to the responsiveness to TRH and also to tumor volumes. A Spearman correlation coefficient was calculated between the tumor volumes and ΔGH_max-min_ as well as peak GH levels during the TST to investigate the relationship between tumor volume and responsiveness to TRH.

### 2.9. Ethics Statement

This study was approved by the institutional review board (IRB) of Kyung Hee University Hospital (IRB number KMC IRB 1333-06). The informed consents from the patients were waived by the boards due to the retrospective design of this study.

## 3. Results

### 3.1. Comparison of TST Results between TRH Responders and Nonresponders

**Figure 1:**
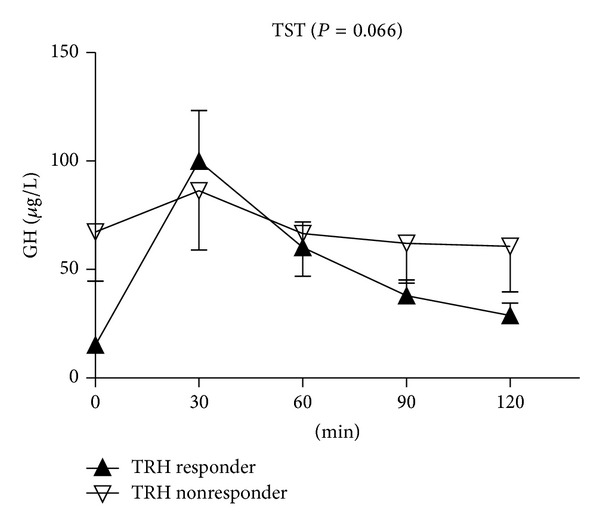
Comparison of GH levels according to the responsiveness to TRH during the entire TST time. Data represent mean ± standard error. RMANOVA was used for the statistical comparison, and Greenhouse-Geisser correction was applied if compound symmetry was not satisfied based upon Mauchly's sphericity test. Abbreviations: GH: growth hormone; TRH: thyrotropin releasing hormone; TST: TRH stimulation test; RMANOVA: repeated measure one-way analysis of variance.

Thirty-two patients (78.0%) were classified as TRH responders and 9 as nonresponders (Table 1). TRH responders were significantly older than nonresponders, but BMI, basal GH, and IGF-I levels did not show significant difference between TRH responders and nonresponders. ΔGH_max-min_ during the TST were significantly higher in TRH responders than in nonresponders (82.0 ± 94.3 versus 33.9 ± 43.1 *μ*g/L, *P* = 0.035 for ΔGH_max-min_). Tumor volumes did not show statistically significant difference according to the responsiveness to TRH. The between-group difference in GH levels remained nonsignificant during the entire TST time between TRH responders and nonresponders (*P* = 0.066, Figure 1). 

### 3.2. Comparison of Results of the TST between Macroadenomas and Microadenomas

**Figure 2:**
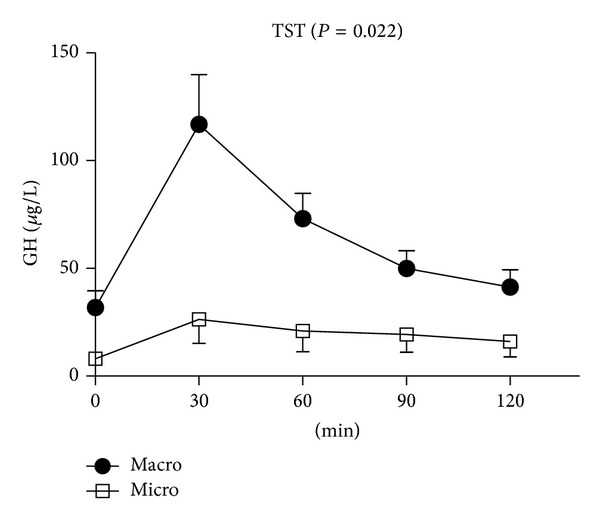
Comparison of GH levels between macroadenomas and microadenomas during the TST. Data represent mean ± standard error. RMANOVA was used for the statistical comparison, and Greenhouse-Geisser correction was applied if compound symmetry was not satisfied based upon Mauchly's sphericity test. Abbreviations: GH: growth hormone; TRH: thyrotropin releasing hormone; TST: TRH stimulation test; macro: macroadenoma; micro: microadenoma; RMANOVA: repeated measure one-way analysis of variance.

Thirty-two patients had macroadenomas (mean volume 6187 ± 14583 mm^3^), and 9 patients had microadenomas (mean volume 175 ± 171 mm^3^) (Table 2). Twenty-five patients with macroadenomas and 7 with microadenomas were classified as TRH responders. BMI did not show any significant differences when compared according to tumor volume. Basal GH levels were significantly higher in those patients with macroadenomas (*P* = 0.012), but IGF-I levels did not show any significant difference according to tumor volume. Both peak GH levels during the TST and ΔGH_max-min_ were higher in macroadenomas than in microadenomas (124.1 ± 129.0 versus 27.5 ± 34.5 *μ*g/L, *P* = 0.002; 90.3 ± 125.8 versus 12.7 ± 22.0 *μ*g/L, *P* = 0.026). When only patients with macroadenomas were analyzed, ΔGH_max-min_ during the TST were significantly higher in TRH responders (105.8 ± 138.1 versus 34.8 ± 30.1 *μ*g/L, *P* = 0.024: *P* value not shown in the table), while tumor volumes were not significantly different. GH levels remained significantly higher in macroadenomas than in microadenomas over the entire TST time (*P* = 0.022, Figure 2). 

### 3.3. Correlation between Results of the TST and Tumor Volume

**Figure 3:**
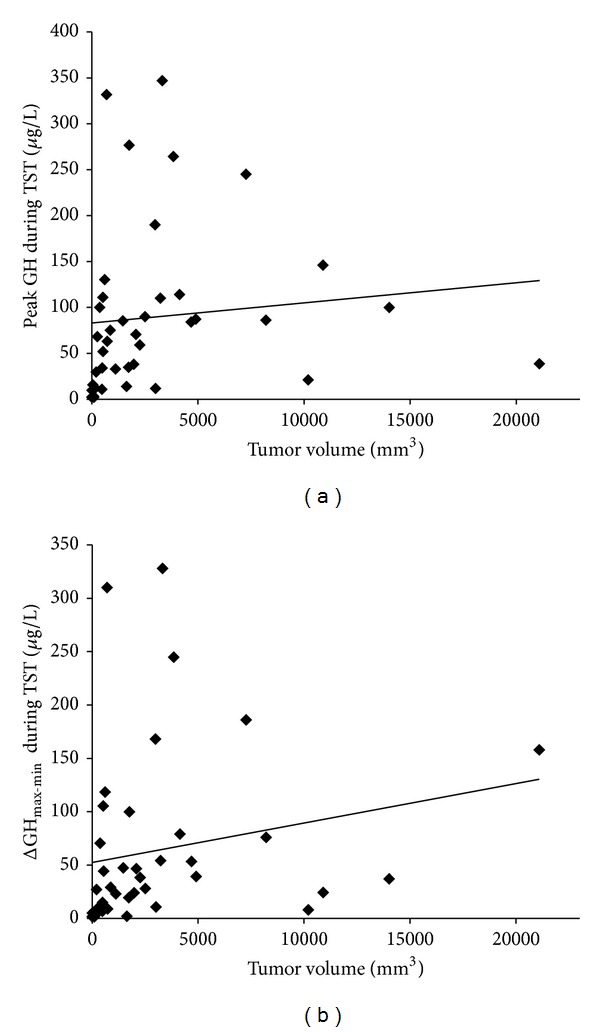
Correlation between the tumor volume and peak GH levels during the TST (*r* = 0.498, *P* = 0.001) (a) and ΔGH_max-min_ during the TST (*r* = 0.420, *P* = 0.006) (b).

The relationship between the peak GH levels during the TST and the tumor volume was investigated by calculating the Spearman correlation coefficient, which revealed a moderate but significant correlation between two parameters (*r* = 0.498, *P* = 0.001, Figure 3(a)). The same analysis between ΔGH_max-min_ during the TST and the tumor volume revealed an analogous result (*r* = 0.420, *P* = 0.006, Figure 3(b)). 

### 3.4. Comparison of GH Suppression during the OST between TRH Responders and Nonresponders

In the OST, TRH responders had a GH suppression rate which was not significantly different from that of TRH nonresponders (89.7 ± 12.4% versus 80.4 ± 19.8%, *P* = 0.136).

## 4. Discussion

The present study investigated the relationship between the volume of GH-producing pituitary adenomas and their responsiveness to TRH. Between TRH responders and nonresponders, basal GH, IGF-I levels, and tumor volume were not significantly different but the between-group difference of GH levels remained near significant over the entire TST time. ΔGH_max-min_ during the TST were significantly different according to the responsiveness to TRH. Peak GH levels and ΔGH_max-min_ during the TST showed significantly positive correlations with tumor volume with higher levels in macroadenomas than in microadenomas. GH levels over the entire TST time also remained significantly higher in macroadenomas than in microadenomas. Altogether, these results showed that responsiveness to TRH during the TST in GH-producing pituitary adenomas was not inversely correlated with tumor volume. Our additional analysis demonstrated that the GH suppression rate after octreotide injection did not differ regardless of responsiveness to TRH. All of these findings are substantially in conflict with previously reported data [10, 16]. 

A number of possible mechanisms for the paradoxical response after TRH administration have been suggested. Locally released TRH produced by pituitary adenomas could act as an autocrine and/or paracrine regulator to affect hormone release or tumor growth [11, 12]. Alternatively, it has been hypothesized that TRH may lead to acute GH release by inhibiting somatostatin release [13], or TRH could be synthesized endogenously by the anterior pituitary gland [14]. Yamada et al. have suggested that dedifferentiation of tumor cells as a result of inappropriate expression of TRH receptors may cause the paradoxical response of GH after TRH administration [15]. The level of TRH receptor 1 (THRH-1) mRNA expression was positively correlated with the responsiveness of GH to TRH administration [22]. Thus, the notion of dedifferentiation of tumor cells could be one of the relevant explanations for paradoxical responses, but still its exact mechanism remains largely unclear. Moreover, few studies have explored the relationship between responsiveness to TRH during the TST and tumor volume in GH-producing pituitary adenomas. De Marinis et al. reported that the preoperative GH paradoxical response to TRH was often present in small pituitary adenomas [10]. Arita et al. similarly reported that the tumors of TRH responders were smaller but their levels of serum GH per volume were higher in TRH nonresponders and the TRH-induced GH response was inversely related to the tumor volume [16]. 

It was reported that patients with GH-producing pituitary adenomas and a tumor-stimulatory G protein (*gsp*) mutation had small tumor volume with a higher rate of TRH responders [17, 18], which provided a theoretical background for inverse correlation between the GH response to TRH and tumor volume [16]. However, a number of previous studies have also reported that pituitary adenomas with *gsp* mutation were rather larger than those without mutation [23, 24]. Therefore, the presence of this mutation may not be sufficient to explain the relationship between responsiveness of GH to TRH and tumor volume. We previously reported that as the tumor becomes larger, there exists a portion of the GH secretion which escaped physiologic regulation by somatostatinergic tone (SST) [25]. One of the known hypotheses for the paradoxical response in GH to TRH administration is the inhibition of somatostatin release by TRH [13]. Thus, unlike the previous findings [10, 16], it can be further hypothesized that these two mechanisms may behave synergistically to result in the increase of GH secretion after TRH administration as the tumor volume increases. Interestingly, our data also showed there was no difference in basal IGF-I levels according to the responsiveness to TRH (Table 1). There was no correlation between IGF-I levels and tumor sizes at diagnosis according to the Liege Acromegaly Survey [26], and this may explain the reason for the absence of a significant relationship between responsiveness to TRH and basal IGF-I levels in newly diagnosed acromegalic patients. 

A previous study also reported that TRH responders showed a higher GH suppression rate than nonresponders during the OST [16]. This result was seemingly supported by preceding reports that pituitary adenomas with the *gsp *oncogene mutation were shown to experience stronger GH suppression by octreotide and pituitary adenomas with *gsp* mutation were more likely to be TRH responders [17, 18]. In contrast, our data failed to demonstrate any significant difference in the GH suppression rate between TRH responders and nonresponders (89.7 ± 12.4% versus 80.4 ± 19.8%, *P* = 0.136). TRH exerts its action through a phosphatidyl inositol-protein kinase pathway [27]. In contrast, octreotide signaling involves various somatostatin receptors, the inhibition of adenylyl cyclase activity, and modulation of the activity of potassium and calcium channels as well as stimulation of phosphotyrosine phosphatase or mitogen activated protein kinase activity [28–31]. These two signaling pathways have not been shown to interact with each other, suggesting that a significant association between the GH response to TRH and octreotide is unlikely.

There are some limitations to this study, including a relatively small number of acromegalic patients with TRH nonresponders. However, the percentage of TRH nonresponders was relatively similar between previously published data (17/62, 27.4%) [16] and ours (9/41, 22.0%, Table 1). Also, there were not many patients with microadenomas, and as a result, it could cause a paradox that most of microadenomas (7/9) but only 78% of macroadenomas were classified as TRH responders (25/32, Table 2), possibly leading to misunderstanding that responsiveness might track with smaller size. It is well known that approximately 70% of GH-producing adenomas are macroadenomas at diagnosis [32], and thus there must be larger prospective studies to solve this limitation. In addition, the histologic characteristics of each tumor were not taken into account in this study.

In conclusion, we expanded the number of patients with newly diagnosed GH-producing pituitary adenomas who had been evaluated with TRH to examine the paradoxical response and concluded that, unlike the previously published data [10, 16], the relationship between the GH response to TRH and tumor volume did not demonstrate any evidence for the inverse correlation; ΔGH_max-min_ during the TST but not basal GH, IGF-I levels, and tumor volume showed significant differences according to responsiveness to TRH and tumor volume. Peak GH during the TST was significantly different according to tumor volume. Both ΔGH_max-min_ during the TST and peak GH during the TST were positively correlated with tumor volumes. The paradoxical response of GH to TRH appears to result from the unpredicted and still unknown interactions of various factors [16, 33], and this may explain why there have been inconsistent results regarding the relationship between the responsiveness to TRH and tumor volume. Additional studies with a larger number of patients are warranted to provide better understanding of the basic characteristics of responsiveness to TRH during the TST in patients with GH-producing pituitary tumors.

## Figures and Tables

**Table 1:** Baseline characteristics of 41 patients with GH-producing pituitary adenomas according to their response patterns to the TRH suppression test.

	TRH responder	TRH nonresponder	*P*
Number of patients	32	9	
Age at diagnosis	45 ± 11	34 ± 8	0.002*
BMI, kg/m^2^	26.3 ± 3.1^a^	27.7 ± 3.8^b^	0.336
Basal GH, *μ*g/L	19.4 ± 28.7	49.3 ± 56.6	0.072
Basal IGF-I, *μ*g/L^a^	890 ± 312^c^	1012 ± 452^d^	0.411
Peak GH during TST, *μ*g/L	99.1 ± 100.4	74.6 ± 70.1	0.740
ΔGH_max⁡-min⁡_ during TST, *μ*g/L	82.0 ± 94.3	33.9 ± 43.1	0.035*
Tumor volume, mm^3^	4886 ± 15854	4834 ± 6338	0.429

Data were expressed as mean ± standard deviation. The Mann-Whitney test was used for the statistical comparison between TRH responders and non-responders.

*P < 0.05.

^a^Data were available in 16 cases.

^b^Data were available in 10 cases.

^c^Data were available in 25 cases.

^d^Data were available in 13 cases.

Abbreviations: TRH: thyrotropin-releasing hormone; BMI: body mass index; GH: growth hormone; IGF-I: insulin-like growth factor-I; TST: TRH stimulation test.

**Table 2:** Baseline characteristics of 41 patients with GH-producing pituitary adenomas according to their tumor sizes.

Responsiveness to TRH	Macroadenomas			Microadenomas			*P* ^¶^
	Responders	Non-responders	Total	Responders	Non-responders	Total	
Number of patients	25	7	32	7	2	9	
Tumor volume, mm^3^	6937 ± 16399	3506 ± 3507	6187 ± 14583	147 ± 138	275 ± 308	175 ± 171	<0.026*
Age at diagnosis	43 ± 12	38 ± 8	42 ± 12	33 ± 9	47 ± 14	36 ± 11	0.181
BMI, kg/ m^2^	25.7 ± 2.9	28.9 ± 3.5	26.8 ± 3.4^a^	27.7 ± 4.1^b^	N/A	27.8 ± 4.1^b^	0.674
Basal GH, *μ*g/ L	23.6 ± 29.7	79.1 ± 71.4	35.8 ± 47.0	10.6 ± 13.9	13.0 ± 8.3	11.2 ± 12.5	0.012*
Basal IGF-I, *μ*g/ L	919 ± 309	1199 ± 551	976 ± 376^c^	750 ± 330	827 ± 78	770 ± 283^d^	0.111
^§^Peak GH during TST, *μ*g/ L	129.0 ± 139.8	105.0 ± 84.4	124.1 ± 129.0	28.2 ± 39.4	24.8 ± 12.9	27.5 ± 34.5	0.002*
^§∗^ΔGH_max⁡−min⁡_ during TST, *μ*g/ L	105.8 ± 138.1	34.8 ± 30.1	90.3 ± 125.8	13.4 ± 25.2	10.3 ± 6.5	12.7 ± 22.0	0.026*

Data were expressed as mean ± standard deviation.

^¶^ *P* values between macroadenomas (*n* = 32) and microadenomas (*n* = 9) were calculated using the Mann-Whitney test.

*P < 0.05.

^§^The Mann-Whitney test only between TRH responders and non-responders with macroadenomas demonstrated the significant difference between the two groups.

^a^Data were available in 22 cases.

^b^Data were available in 4 cases.

^c^Data were available in 30 cases.

^d^Data were available in 8 cases.
